# Mucin-1 Gene Mutation and the Kidney: The Link between Autosomal Dominant Tubulointerstitial Kidney Disease and Focal and Segmental Glomerulosclerosis

**DOI:** 10.1155/2018/9514917

**Published:** 2018-07-31

**Authors:** H. Trimarchi, M. Paulero, T. Rengel, I. González-Hoyos, M. Forrester, F. Lombi, V. Pomeranz, R. Iriarte, A. Iotti

**Affiliations:** ^1^Nephrology Service, Hospital Británico de Buenos Aires, Buenos Aires, Argentina; ^2^Pathology Service, Hospital Británico de Buenos Aires, Buenos Aires, Argentina

## Abstract

Glomerular diseases are one of the most frequent causes of chronic kidney disease, focal and segmental glomerulosclerosis being one of the commonest glomerulopathies. However, the etiology of this glomerular entity, which merely depicts a morphologic pattern of disease, is often not established and, in most of the patients, remains unknown. Nephrologists tend to assume focal and segmental glomerulosclerosis as a definitive diagnosis. However, despite the increasing knowledge developed in the field, genetic causes of glomerular diseases are currently identified in fewer than 10% of chronic kidney disease subjects. Moreover, unexplained familial clustering among dialysis patients suggests that genetic causes may be underrecognized. Secondary focal and segmental glomerulosclerosis due to genetic mutations mainly located in the podocyte and slit diaphragm can occur from childbirth to adulthood with different clinical presentations, ranging from mild proteinuria and normal renal function to nephrotic syndrome and renal failure. However, this histopathological pattern can also be due to primary defects outside the glomerulus. The present report illustrates an adult case of secondary focal and segmental glomerulosclerosis with a dominant tubulointerstitial damage that led to the pursue of its cause at the tubular level. In this patient with an undiagnosed family history of adult kidney disease, a genetic study unraveled a mutation in the mucin-1 gene and a final diagnosis of adult dominant tubular kidney disease-MUC1 was made.

## 1. Introduction

Focal and segmental glomerulosclerosis (FSGS) is classified into primary and secondary causes [1]. While the former is mainly due to still unidentified controversial circulating permeability factors and presents clinically with severe nephrotic syndrome and a grim prognosis, the latter may be due to numerous widely dissimilar etiologies with completely different pathophysiological mechanisms [1, 2]. In general, secondary causes of focal and segmental glomerulosclerosis present with lower levels of proteinuria and a slow decline in kidney function. Regardless of the cause, the main concern that encompasses focal and segmental glomerulosclerosis is the fact that it refers just to a morphological pattern of disease [1]. Finally, in general nephrologists tend to accept FSGS as a definitive diagnosis and mainly focus on the management of the clinical markers of kidney disease progression, as proteinuria and hypertension. Rarely do nephrologists search for the genetic causes that may cause FSGS and assume it to be of glomerular origin when evident causes of its secondary essence have been discarded, as HIV infection, ureteral-vesical reflux, obesity, pharmacological causes, hyperfiltration due to a reduction in the glomerular mass, cocaine abuse, and sickle cell disease, among others [1, 2]. Interestingly, the usually nonnephrotic range of proteinuria cannot differentiate between FSGS due to podocyte mutations from the other causes. In this regard, the clinical background, renal imaging, and certain histopathological features encountered in the kidney biopsy can guide or suggest a possible etiology. In this case report, the coexistence of glomerulosclerosis and relevant tubulointerstitial damage with mononuclear infiltration suggested an extraglomerular origin of FSGS. The unexpected diagnosis of a mutation in the mucin gene (MUC-1) was made and remarks how a tubular derangement can cause secondary glomerular damage as glomerulosclerosis, underscoring that FSGS is a pattern of disease.

## 2. Case Presentation

**Figure 1:**
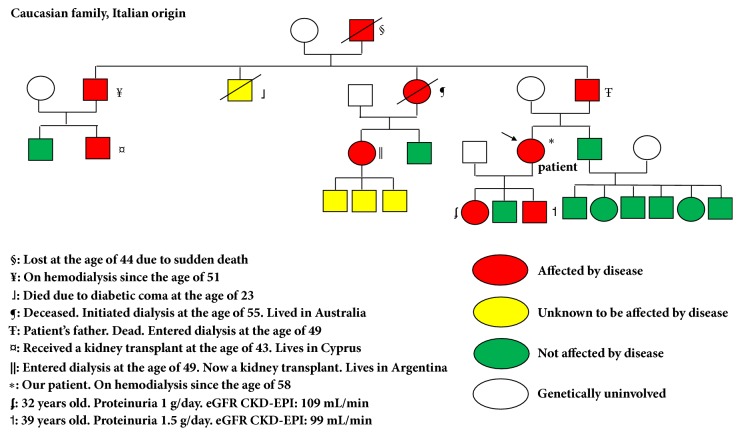
Family tree.

**Figure 2:**
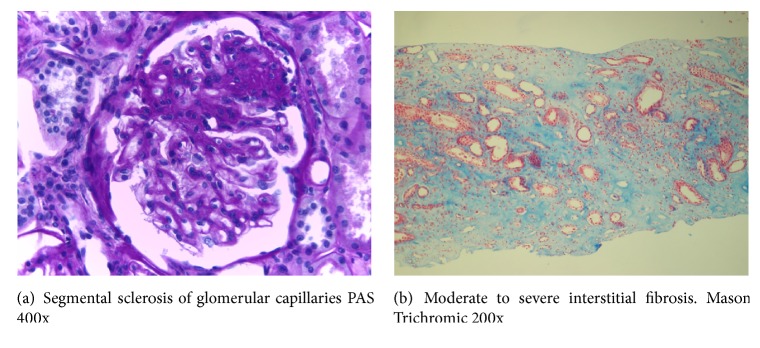


A 56-year-old female was referred to the nephrologist due to apparently chronic kidney disease (CKD), diagnosed on a routine laboratory check-up. The patient was asymptomatic, Past medical record was contributory for three normal pregnancies. There was no background of alcohol intake, tobacco consumption, drug abuse, or medication exposure. There was a family history of CKD (Figure 1). Physical examination was unremarkable. Abnormal blood tests were as follows: Haematocrit 37%; haemoglobin 11.9 g/dL; bicarbonate 21 mEq/L; urea 78 mg/dL (normal value 20-50 mg/dL); serum creatinine 2 mg /dL; uric acid 6.4 mg/dL; creatinine clearance 42 ml/min; proteinuria 0.2 g/day; urinary sodium excretion 188 mEq/day; urine pH: 6, urinary density 1015. Urinary sediment was unremarkable. HIV, HCV, and HBV were negative; C3, C4, and CH50 were within normal limits. ANA, p-ANCA, c-ANCA, antiglomerular basement membrane antibody, and antiphospholipid antibodies were reported as negative. Renal sonogram disclosed two kidneys, normal in shape and size. A kidney biopsy was performed. Light microscopy disclosed 30 glomeruli: 6 completely obliterated, 8 presented peripheral sclerosis of the glomerular tuft with adhesions between parietal and visceral epithelial cells of Bowman's capsule, and 6 depicted mild mesangial expansion (Figure 2). Tubular atrophy and interstitial fibrosis were 30%. Blood vessels showed mild intimal sclerosis in arterioles. Immunofluorescence was negative. Electron microscopy: diffuse effacement of podocyte foot processes existed with microvillous transformation. Basal membrane was normal. Tubules were normal. Pathology report was as follows: focal and segmental glomerulosclerosis with moderate interstitial fibrosis and tubular atrophy. Patient was started on enalapril 5 mg twice a day and simvastatin 10 mg/day and on appropriate diet.

She was lost to follow-up. Sixteen months later the patient returned to the nephrologist due to asthenia, fatigue, and cramps. Blood pressure was 110/70 mmHg. Significant blood test results were as follows: Haematocrit 32%; haemoglobin 9.2 g/dL; potassium 5.5 mEq/L; bicarbonate 19 mEq/L; serum calcium 9.5 mg/dL; serum phosphate 6.2 mg/dL; serum magnesium 2.2 mg/dL; urea 111 mg/dL; serum creatinine 3.78 mg/dL; uric acid 8.1 mg/dL; albumin 4.3 g/dL; creatinine clearance 21 ml/min; proteinuria 0.29 g/day; urinalysis was unremarkable. Urine pH was 7 and urinary density 1010. A renal magnetic resonance imaging was noncontributory. The patient was prescribed erythropoietin 2000 U every other day, enalapril 5 mg bid, calcium carbonate 2 g/day, sodium bicarbonate two tea spoons daily, and polystyrene calcium sulfonate. Six months later the patient was started on hemodialysis (creatinine clearance 12 mL/min). A genetic study disclosed the insertion of a cytosine nucleotide in the VNTR (Variable Number Tandem Repeats) region of the MUC-1 gene, consistent with a mutation of the mucin-1 gene previously reported [3]: cDNA NM_001204286.1, protein NP_001191215.1, SNaPshot. The diagnosis of ADTKD-MUC1 (Autosomal Dominant Tubulointerstial Kidney Disease-Mucin-1) was finally made. The laboratory results of the patient's daughter revealed mild proteinuria and normal kidney function (Figure 1). A kidney biopsy revealed mild tubulointerstitial disease and focal and segmental glomerulosclerosis in 2 out of 16 glomeruli. She was started on enalapril and nephroprotection and genetic counseling was given to her.

## 3. Discussion

Our patient presented with a long lasting undiagnosed family history of chronic kidney disease (CKD) while some of her relatives had progressed to end-stage kidney disease as adults. As the clinical case was assumed as idiopathic chronic kidney disease, the kidney biopsy was considered mandatory. Moreover, the family background showed a slow but progressive trend of the disease that urged to obtain tissue promptly, as the benefits of performing a biopsy with diagnostic purposes is lower at advanced stages of CKD. In our opinion, as long-term proteinuria was mild, a primary glomerulopathy appeared unlikely. However, the pathology report informed that FSGS dominated the glomerular architecture. This was accompanied by moderate interstitial fibrosis, tubular atrophy, and interstitial inflammation, which were in accordance with the glomerular level of compromise and also with the clinical picture. Therefore, a secondary cause of FSGS was pursued. The concomitant occurrence of FSGS in a case of ADTKD, as previously reported [4], could be a histological pattern of injury secondary to the common end result of many chronic kidney conditions, in a nonspecific manner, and not as a direct cause of FSGS by the MUC-1 mutation.

The family tree suggested an autosomal dominant pattern of inheritance. In addition, the middle-age adult onset on chronic kidney insufficiency and end-stage kidney disease was an important aspect to take into consideration. Noteworthily, hypertension was not present either at stage 3 of CKD (at the time of diagnosis) or when the patient entered dialysis. Finally, both a low-grade proteinuria plus the medullar histologic findings were indicating a genetic tubular cause was to be ruled out. In this regard, an autosomal dominant tubulointerstitial kidney disease was taken into consideration [4–6].

Autosomal dominant tubulointerstitial kidney disease is a rare entity and is subclassified on a genetic basis that encompasses four mutations in the genes encoding uromodulin (UMOD), hepatocyte nuclear factor 1-*β* (HNF 1B), renin (REN), and mucin-1 (MUC-1) [4]. This novel and practical classification replaces cumbersome and confusing previous ones and suggests straightforward diagnostic criteria. Most of the clinical and histologic findings are nonspecific. As remarked in the KDIGO guidelines, there is usually a known familial history of kidney disease, and some members may have not been diagnosed properly due to death even before CKD symptoms arise, as it was the case in our patient's father [4]. The average age of renal replacement therapy entrance is between 40 and 60 years, although this may depend on other variables as degree of penetrance of the mutation, hyperuricemia, and comorbidities [4–6]. Hypertension is typically absent in these subjects, while cysts are not predominant, although they can be more frequently encountered at advanced stages of these diseases, consequently not contributing to renal damage [4]. Due to their rarity, the prevalence and incidence of the different types of ADTKD remains unknown [4]. The main features although not exclusive of the four types are depicted in Table 1. Briefly, in ADTKD-UMOD, hyperuricemia and gout appear most frequently in adulthood and cysts are not frequent, but if present they tend to be cortical [4, 7, 8]. In ADTKD-REN, anemia (which resolves in puberty) and hypotension are present at childhood, while hyperuricemia and hyperkalemia are distinguishing features [4, 9]. In ADTKD-HNF1B, diabetes mellitus, pancreatic atrophy, and urogenital abnormalities are present, together with hypomagnesemia, hypokalemia, and liver function test abnormalities [10]. Finally, in ADTKD-MUC1, there are occasional cortical cysts and no other main characteristics [3, 4]. It may be presumed that the abnormal secreted mucin protein plugs in the distal tubule and causes an increase in the intraluminal pressure of the tubules, behaving as a postrenal cause of CKD. In this setting, this chronic situation impedes a normal clearance at the glomerular filtration barrier, leading to inflammation and glomerulosclerosis, as found in our patient (Figure 2). As mentioned above, the interstitial inflammation and tubular atrophy tend to dominate the biopsy picture. In the case of ADTKD-MUC1, distal tubular intracellular accumulation of the abnormal codified peptide named mucin fs can be identified for research purposes [4]. Histologically, FSGS has been described as a nonspecific finding in kidney biopsies of patients with ADTKD [4, 8, 11] (Table 1).

Mucins are highly molecular heavily glycosylated transmembrane proteins classified as secretory or membrane-bound. MUC1 is a membrane-bound mucin with a high expression throughout the distal nephron and is involved in the protection and lubrication of the distal tubular lumen [12, 13]. In addition, as a transmembrane protein it is involved in many intracellular functions, particularly in signal transduction [12, 13]. Although genetic testing for UMOD, REN, and HNF1B mutations is well established, MUC1 genetic testing remains challenging [11]. Finally, there is no specific therapy for this disease. In ADTKD, diuretics should be used with caution or avoided, as they may aggravate hyperuricemia and volumen depletion [14]. Liberal water intake is recommended to compensate for possible urinary concentration defects. Nonsteroidal anti-inflammatory drugs should be avoided [4].

In conclusion, an initially diagnosed case of end-stage renal disease due to focal and segmental glomerulosclerosis with moderate tubulointerstitial compromise was later encountered to be secondary to a rare genetic mutation in mucin at the tubular level, known as ADTKD-MUC1.

## Figures and Tables

**Table 1:** Main findings of the different genetic subtypes of ADTKD.

	UMOD	MUC1	REN	HNF 1*β*
Clinical findings	Gout Occasional cortical cysts	Occasional cortical cysts	Hypotension Anemia	Diabetes mellitus Pancreatic atrophy Urogenital anomalies

Age at presentation	adulthood	adulthood	childhood	Early childhood

Laboratory findings	Hyperuricemia	No characteristic findings	Hyperuricemia Hyperkalemia	Hypomagnesemia Hypokalemia Elevated liver enzyme levels

Pathology findings	Tubulointerstitial damage. Secondary FSGS Intracellular UMOD deposits in Thick Ascending Henle's limbs	Tubulointerstitial damage. Secondary FSGS Intracellular accumulation of MUC1 in distal tubules	Tubulointerstitial damage. Secondary FSGS	

ADTKD, Adult dominant tubulointerstitial kidney disease; UMOD, uromodulin; MUC1, mucin-1; REN, renin; HNF 1*β*, Hepatocyte nuclear factor 1*β*; FSGS, focal and segmental glomerulosclerosis.
